# Cell wall methanol as a signal in plant immunity

**DOI:** 10.3389/fpls.2014.00101

**Published:** 2014-03-18

**Authors:** Tatiana V. Komarova, Ekaterina V. Sheshukova, Yuri L. Dorokhov

**Affiliations:** ^1^A. N. Belozersky Institute of Physico-Chemical Biology, Moscow State UniversityMoscow, Russia; ^2^N. I. Vavilov Institute of General Genetics, Russian Academy of ScienceMoscow, Russia

**Keywords:** cell wall, methanol, pectin, pectin methylesterase, plant immunity, priming

## Abstract

Cell wall pectin forms a matrix around the cellulose–xyloglucan network that is composed of rhamnogalacturonan I, rhamnogalacturonan II, and homogalacturonan (HG), a major pectic polymer consisting of α-1,4-linked galacturonic acids. HG is secreted in a highly methyl-esterified form and selectively de-methyl-esterified by pectin methylesterases (PMEs) during cell growth and pathogen attack. The mechanical damage that often precedes the penetration of the leaf by a pathogen promotes the activation of PME, which in turn leads to the emission of methanol (MeOH), an abundant volatile organic compound, which is quickly perceived by the intact leaves of the damaged plant, and the neighboring plants. The exposure to MeOH may result in a “priming” effect on intact leaves, setting the stage for the within-plant, and neighboring plant immunity. The emission of MeOH by a wounded plant enhances the resistance of the non-wounded, neighboring “receiver” plants to bacterial pathogens and promotes cell-to-cell communication that facilitates the spread of viruses in neighboring plants.

## INTRODUCTION

Plant cells are covered with a dense extracellular matrix that prevents direct contact between adjacent cells and pathogens. Damage to the plant epidermis caused by abiotic (wind, hail, and rain) and biotic (insects) factors may allow the penetration of pathogens (bacteria, fungi, oomycetes, and nematodes) into the intercellular space of the leaf and virus particles into the cell. Thus, plant wounding is one of the conditions for pathogen entry. However, mechanical damage to the leaf promotes the emission of volatile organic compounds (VOCs), including the green leaf volatiles (GLVs), and methanol (MeOH), which are quickly perceived by the intact leaves of the damaged plant and the intact neighboring plants (Dorokhov et al., 2014). The transport of VOCs is much faster compared to the transport of infectious viral entities and bacterial effectors through the phloem. Thus, exposure to VOCs may result in a “priming” effect on intact leaves, setting the stage for subsequent plant immunity. GLVs are associated with the smell of a freshly mown lawn and are derived from C18 fatty acids released from damaged membranes. MeOH, quantitatively the most important plant volatile after CO_2_, is a product of the demethylation of pectin by the pectin methylesterases (PMEs) during cell wall (CW) formation and modification (Pelloux et al., 2007).

## PME-MEDIATED PLANT IMMUNITY

Cell wall pectin forms a matrix around the cellulose–xyloglucan network that is composed of three main components called rhamnogalacturonan I (RGI), rhamnogalacturonan II (RGA II), and homogalacturonan (HG), a major pectic polymer consisting of α-1,4-linked galacturonic acids (Peaucelle et al., 2012). HG is secreted in a highly methyl-esterified form and selectively de-methyl-esterified by PMEs, resulting in MeOH formation. The *PME* genes encode a pro-PME precursor with an *N*-terminal extension of variable length that is essential for protein targeting to the endoplasmic reticulum (Dorokhov et al., 1999). PME maturation requires removal of the PME leader including the transmembrane domain and spacer sequence (Dorokhov et al., 2006a). It was hypothesized that the spacer sequence plays a role in subcellular targeting and acts as an intramolecular chaperone for folding of the mature enzyme or as an autoinhibitor during transport through the endomembrane system (Pelloux et al., 2007). PME participates in CW modulation during general plant growth as it is involved in cell expansion and CW modification (Pelletier et al., 2010). The synthesis of PME is one of the aspects of plant growth that leads to the demethylesterification of the elastic “soft” pectins that accompanies MeOH generation (Komarova et al., 2014) as part of the natural division and maturation of the plant cell. After demethylesterification, pectate can form Ca^2^^+^-pectate cross-linked complexes of rigid “hard” pectin, referred to as “egg boxes” (Peaucelle et al., 2012).

The important role of PME in the resistance of plants to fungi and bacteria has been demonstrated (Pelloux et al., 2007). A higher degree of pectin methyl esterification in certain plants induces resistance to pathogenic fungi (Lionetti et al., 2012). CW pectin methyl esterification may have an impact on plant resistance because highly methyl-esterified pectin can be less susceptible to hydrolysis by pectic enzymes such as fungal endopolygalacturonases. This view is supported by experiments performed with plants that were stably transformed with the *PME inhibitor (PMEI) *gene. The PMEI transgenic *Arabidopsis* (Lionetti et al., 2007) and durum wheat (Volpi et al., 2011) plants exhibited high levels of resistance to fungal and bacterial pathogens. Moreover, PME-mediated pectin methyl de-esterification may influence the polygalacturonase-mediated release of pectin-derived compounds, which in turn elicits a defense response (Pelloux et al., 2007; Lionetti et al., 2012).

The role of PME in viral infection is more complicated. PME interacts with the movement protein (MP) of the *Tobacco mosaic virus* (TMV; Dorokhov et al., 1999; Chen et al., 2000), suggesting that PME may be involved in the cell-to-cell movement of plant viruses (Chen and Citovsky, 2003). Interestingly, PMEI also interacts with PME to negatively affect viral infection (Lionetti et al., 2014), most likely by interfering with PME and TMV MP binding. The complex role of PME in viral infection is also underscored by the effects of PME on nuclear protein transport (Komarova et al., 2011) and gene silencing mediated by the activation of siRNA and miRNA production (Dorokhov et al., 2006b).

## MeOH AND PLANT IMMUNITY

The PME-mediated conversion of HG methoxyl groups into carboxyl groups results in MeOH release. In humans, MeOH is considered to be a poison because alcohol dehydrogenase metabolizes MeOH into toxic formaldehyde. However, recent data have indicated that MeOH is actually a naturally occurring compound in normal, healthy human individuals. MeOH is not toxic to plant cells and has long been assumed to be a metabolic waste product. Recently, it has been shown that MeOH may regulate plant growth (Komarova et al., 2014) and serve an alarm function (Dorokhov et al., 2012a). The effects of PME-generated MeOH emitted from plants (“emitters”) on the defensive reactions of other plants (“receivers”) were studied (Dorokhov et al., 2012a). The results of this study led to the conclusion that MeOH is a signaling molecule that is involved in within-plant and plant-to-plant communication (Dorokhov et al., 2012a).

Mechanical damage to plants drastically increases MeOH and GLVs emission. GLVs that are rapidly released from wounded leaves may in turn stimulate PME-generated MeOH production (Dorokhov et al., 2012a). Herbivore attacks also increase MeOH emission levels: *Manduca sexta *caterpillars enhance wound-induced MeOH emission in *Nicotiana attenuate* (von Dahl et al., 2006). The over-expression of PME, derived from *Arabidopsis thaliana *and *Aspergillus niger*, in transgenic tobacco plants enhances resistance to polyphagous insect pests (Dixit et al., 2013). Transgenic plants with a silenced *PME* gene exhibited a 50% reduction in PME activity in their leaves and a 70% reduction in herbivore-induced MeOH emissions compared to wild type plants. This result demonstrates that herbivore-induced MeOH emissions originate from pectin demethylation by PME (Körner et al., 2009). The emission of MeOH is very fast and can be detected immediately following mechanical damage. Thus, the MeOH emitted from wounded leaves is produced by two forms of PME: pre-existing PME deposited in the CW before wounding, which allows rapid MeOH release (Körner et al., 2009), and PME that is synthesized *de novo* after wounding (Dorokhov et al., 2012a), which likely generates MeOH for an extended period.

Unlike longer-chain alcohols, the MeOH emitted by a wounded plant attracts insects and bark beetles. Moreover, mice prefer the odor of MeOH to the odors of other plant volatiles under laboratory conditions, and MeOH exposure alters the accumulation of mRNA in the mouse brain (Dorokhov et al., 2012b). This finding led to the conclusion that the MeOH emitted by wounded plants may have a role in plant-animal signaling.

Investigations demonstrated (Dorokhov et al., 2012a) that increased MeOH emissions from *PME*-transgenic or mechanically wounded non-transgenic plants retarded the growth of the bacterial pathogen *Ralstonia solanacearum *in neighboring “receiver” plants. The suppression of *R. solanacearum* growth observed in the “receiver” plants could be caused by gaseous MeOH or/and by GLVs. Indeed, *cis*-3-hexen-1-ol evaporated in a desiccator also resulted in decreased bacterial growth in the target plants. However, GLVs rapidly released from wounded leaves stimulated PME-generated MeOH production (Dorokhov et al., 2012a), suggesting that their influence on bacterial growth may be indirect. MeOH-stimulated antibacterial resistance was preceded by the upregulation of genes that control stress response and cell-to-cell communication in the “receiver”. Antibacterial resistance accompanied by MeOH-induced genes (MIGs) upregulation was most likely related to the transcriptional induction of the *type II proteinase inhibitor (PI-II)* gene. PI-IIs are powerful inhibitors of serine endopeptidases in animals and microorganisms (Turra and Lorito, 2011). The *PI-II *gene is not expressed in the leaves of healthy plants, but it is induced in leaves that have been subjected to different types of stress, including wounding and bacterial infection. *PME*-transgenic tobacco with high levels of *PI-II *expression exhibited increased resistance to *R. solanacearum *(Dorokhov et al., 2012a). This finding supports the role of *PI-II* in the suppression of bacterial proteases.

**FIGURE 1:**
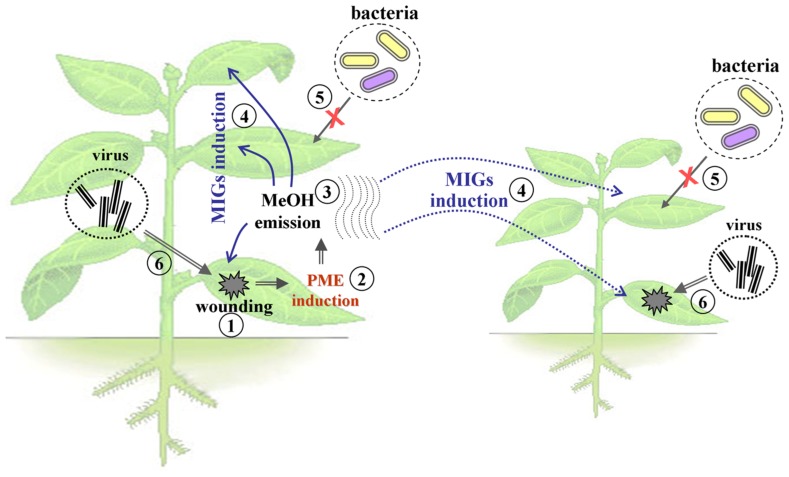
**The model of the effects of methanol emitted by the damaged plant.** Mechanical damage to the leaves of the plant (1), leads to an increase in the expression level of PME (2), and induction of the release of gaseous MeOH (3). Emitted MeOH causes priming of adjacent leaves and neighboring plants, including activation of the MIGs (4), the emergence of bacterial immunity (5), the opening of plasmodesmata, and increased sensitivity to the virus penetration and intercellular distribution of viral pathogens (6).

Experiments with gaseous MeOH provided examples of priming in intact plants (**Figure 1**), which led to conditions conducive for viral infection (Dorokhov et al., 2012a). This effect could be explained by the enhancement of cell-to-cell communication by the MIGs, such as *β-1,3-glucanase* (*BG*; Zavaliev et al., 2011) and *non-cell-autonomous pathway protein* (*NCAPP*; Lee et al., 2003).

A model (**Figure 1**) proposing that MeOH-triggered PD dilation should enhance viral spread within the plant was confirmed in experiments in which BG and NCAPP activated cell-to-cell communication and TMV RNA accumulation. Moreover, gaseous MeOH or the vapors from wounded plants increased TMV reproduction in the “receivers” (Dorokhov et al., 2012a).

Thus, MeOH has a contradictory effect on the sensitivity of the leaves of the “receiver” plant to bacteria and viruses. The mechanisms that underlie this phenomenon are not clear; however, we can consider two factors that may explain this inconsistency in the MeOH-induced effects. First, there is a fundamental difference between bacteria and viruses with respect to their modes of intercellular transport. Bacterial pathogens do not cross the plant CW boundaries because they inhabit the intercellular spaces. In contrast, viral pathogens require cell-to-cell movement for local and systemic spread. Second, the most abundant MIGs can be divided into two groups according to their ability to participate in either bacterial or viral pathogenesis. The first, including *PI-II* and *PME* inhibitor, are involved in immunity against non-viral pathogens. The second group of genes, including *NCAPP* and *MIG-21* (Dorokhov et al., 2012a), is involved in the PD-mediated intercellular transport and reproduction of viruses. The most abundant MIG, the *BG* gene, is involved in antibacterial immunity; however, the BG protein also accelerates PD-mediated intercellular transport.

## CONCLUSION

Based on the available data, we can conclude that wounding-stimulated MeOH that is released into the air by damaged plants or plants compromised by herbivorous insects serves as an alarm to help neighboring plants or adjacent leaves prepare for a defense. The MeOH provides protection against herbivorous insects and plant pathogens such as bacteria. However, considering the role of MeOH in the relationship between viruses and plants, we do not find a negative, or even a neutral, influence of MeOH on viruses. On the contrary, the findings described (Dorokhov et al., 2012a) indicate that MeOH sensitizes the plant to allow the entry and spread of a virus through the plant and between plants by insect vectors. Therefore, MeOH promotes viral propagation. The positive impact of MeOH on viral infection may be explained by several factors. First, plant viruses differ from other types of pathogens as they inhabit the symplast. Furthermore, the survival of a virus depends on its ability to move from cell-to-cell exploiting PD to accumulate to sufficient levels and in enough tissues to guarantee survival despite using a very limited amount of genetic material. Thus, a virus, with its small but highly variable genome, spends its entire life in the cell symplast, while other pathogens occupy the apoplast. Second, the symplast is not only the space in which viruses reproduce, but it is also the site of RNA interference mechanisms that serve to eliminate foreign RNA. The specific degradation of RNA by RNA interference allows the host plant to effectively control viruses and other pathogens. It is known that the intracellular and intercellular transport of silencing factors is necessary for effective RNA interference. Therefore, a MeOH-mediated increase in viral replication may be regarded as compensation for the acquisition of antimicrobial resistance.

## Conflict of Interest Statement

The authors declare that the research was conducted in the absence of any commercial or financial relationships that could be construed as a potential conflict of interest.
